# Synergy of oncolytic adenovirus and immune checkpoint inhibitors: transforming cancer immunotherapy paradigms

**DOI:** 10.3389/fimmu.2025.1610858

**Published:** 2025-07-08

**Authors:** Chong Cheng, Qingzhe Wang, Shuang Zhang

**Affiliations:** Department of Biotherapy, West China Hospital, Sichuan University, Chengdu, Sichuan, China

**Keywords:** oncolytic adenovirus, immune checkpoint inhibitors, drug resistance, tumor microenvironment, immunotherapy

## Abstract

Oncolytic viruses (OVs) offer a promising antitumor strategy by selectively lysing tumor cells and simultaneously activating innate and adaptive immune responses. Recent studies have shed light on the immunostimulatory mechanisms of OVs, particularly oncolytic adenovirus (OAds), which are emerging as leading candidates due to their favorable safety profile, genomic stability, and efficient transduction capacity. Despite the significant progress made by immune checkpoint inhibitors (ICIs) in antitumor therapy, treatment resistance continues to be a major barrier to their clinical effectiveness. OVs and ICIs work synergistically: OVs reprogram the immunosuppressive tumor microenvironment (TME) through immune cell recruitment and pro-inflammatory cytokine production, potentially overcoming ICI resistance. In turn, ICIs enhance T cell function by blocking inhibitory signaling pathways. This review highlights recent preclinical and clinical advancements in the therapeutic potential of combining OAds with ICIs, while also addressing critical translational challenges. We propose a strategic framework for optimizing the development and clinical trial design of these combination therapies to advance precision immunotherapy.

## 1 Introduction

Over the past decade, Immune checkpoint inhibitors (ICIs) have significantly improved the treatment of melanoma and non-small cell lung cancer (NSCLC). Despite their success, challenges such as primary and acquired resistance continue to persist in clinical practice (1–4). The primary resistance mechanisms are largely attributed to the development of an immunosuppressive tumor microenvironment (TME), which is characterized by limited T cell infiltration in “cold” tumors, compensatory activation of immune checkpoints, and metabolic reprogramming of tumor cells (5). To overcome these challenges, research efforts have increasingly centered on the combination of ICIs with other immune-modulatory therapies.

Genetically engineered oncolytic viruses (OVs) have emerged as promising candidates for cancer therapy due to their dual mechanism of action. These viruses selectively target tumor cells, induce immunogenic cell death (ICD), release tumor-associated antigens (TAAs), and activate systemic antitumor immune responses (6). Oncolytic adenovirus (OAds), in particular, have demonstrated significant clinical potential. The recombinant adenovirus H101 was approved in China in 2005 for the treatment of nasopharyngeal carcinoma (7). Second-generation OAds, such as DNX-2401 (Delta-24-RGD), ONCOS-102 (Ad5/3-D24-GM-CSF), and Enadenotucirev (formerly Colad1), have shown promising therapeutic effects in clinical trials (8–10). Despite these advances, the clinical application of OVs faces several challenges, including limitations in the route of administration and the antiviral immune response, which can diminish their efficacy. Intratumoral (IT) administration remain the primary method in clinical trials, while intravenous (IV) administration, which is more relevant to clinical practice, is hindered by neutralizing factors in the blood (11). Most studies have concluded that the antiviral response negatively impacts the antitumor response; however, there are also opposing viewpoints that challenge this notion. Therefore, finding a way to balance both responses is an important aspect that requires further consideration. These challenges have also spurred the development of various OV modifications and novel delivery systems to improve clinical translation.

A growing body of preclinical and clinical evidence indicates that combination therapies outperform monotherapies in terms of tumor control and survival rates (12–15). This advantage stems from the complementary mechanisms of OVs, which remodel the immunosuppressive TME, and ICIs, which alleviate T cell exhaustion and amplify the antitumor immune response. In the treatment with ICIs, the polymorphism of Fcγ receptors can modify the antibody-mediated immune response, thereby influencing the extent of immune system activation (16). It may also result in the immune system attacking normal tissues, especially in the presence of Fcγ receptor variations, which can exacerbate the occurrence of immune-related adverse events (irAEs) (17). OVs have the ability to deliver immunomodulators directly into the TME. Xie et al. engineered OAds to carry transgenes encoding the extracellular domains of SIRPα or Siglec10 on the Fc scaffold to specifically target macrophages, or to encode the extracellular domain of TIGIT to target T cells (18). This method precisely activates specific immune cells. Moreover, OVs can also deliver an anti-PD-1 antibody or a CTLA4-specific single-chain variable fragment (ScFv), thereby further boosting antitumor immune responses with minimal systemic toxicity (19, 20). Currently, two primary strategies for combining OVs and ICIs are being explored: one approach administers OVs and ICIs as separate agents, while the other involves encoding immunomodulatory molecules into the OV genome for local expression. A systematic comparison of these strategies and an in-depth discussion are provided in the study by Wan et al. (21).

This review aims to provide a comprehensive evaluation of the molecular mechanisms, clinical advancements, and challenges associated with OAd-ICI combination therapies. Our focus will be on three primary areas: the immunological basis of their synergistic effects, the key findings from current clinical trials, and the factors that limit the efficacy of these therapies, along with potential solutions. We intend for this review to establish a solid foundation and offer valuable insights to guide future research and optimize clinical trial designs.

## 2 OAds

OVs target tumor cells through receptor-mediated entry. Once inside the cells, the viral genome is transported to the nucleus, where it initiates replication and transcription. As the viral progeny accumulates, they trigger cell lysis and death, a process known as direct oncolysis. This cytolytic action leads to two main outcomes: the release of progeny viruses, which propagate the infection to neighboring tumor cells and the release of TAAs, which enhance antigen presentation by dendritic cells (DCs). This subsequently activates tumor-specific T cell responses, thereby establishing systemic antitumor immunity. Genetically modified OVs, such as those incorporating cytokine genes or modulating immune checkpoints, can further augment these therapeutic effects.

### 2.1 Direct oncolysis

OAds are double-stranded DNA viruses that replicate within the nucleus of host cells. Tumor-specific regulatory elements, such as modified E1A promoters, are employed to restrict viral replication to tumor cells, thereby enhancing the safety of high dose administrations (22–24). The mechanism of viral entry varies among different OAd serotypes. Most OAds utilize the coxsackievirus-adenovirus receptor (CAR), while certain subgroups, such as subgroup B and specific D variants, employ CD46 for cell attachment (25). After internalization through receptor-mediated endocytosis, uncoating proteins disrupt the endosomal membrane, enabling the virus to escape into the cytoplasm. The viral genomes are then transported along microtubules to the nuclear pore complex, where they undergo nuclear translocation (26).

The E1A and E1B genes are essential for initiating viral replication. Specifically, the conserved region 2 (CR2) of E1A interacts with the retinoblastoma (Rb) protein, leading to the release of the E2F transcription factor, which drives the progression of the cell cycle into the S phase—a key event for viral replication in tumor cells (27, 28). Additionally, the E1B-encoded proteins, including the 19 kDa and 55 kDa isoforms, inhibit apoptosis and facilitate viral DNA replication (29, 30). These molecular interactions enhance the tumor specificity of the virus while minimizing off-target toxicity in preclinical models.

ONYX-015, the first tumor-selective adenovirus to undergo clinical evaluation, carries a deletion in the E1B-55K gene, which limits replication to p53-deficient tumor cells while sparing normal cells with intact p53 (31). H101, a derivative of ONYX-015 that also contains a deletion in the E1B/E3 region, was approved by the Chinese FDA as the first commercially available OV (32, 33). DNX-2401 employs an RGD peptide-modified fibronectin to facilitate viral entry through αvβ3/αvβ5 integrins, effectively bypassing the need for CAR receptors in tumor cells (34, 35). Enadenotucirev utilizes a chimeric Ad11p/Ad3 backbone to overcome pre-existing humoral immunity, featuring a unique serotype profile that enhances tumor-selective lysis and fosters immune cell infiltration, establishing it as a highly adaptable therapeutic platform (36–38). A summary of other genetically modified adenoviruses is provided in **Table 1**.

**Table 1:** Genetic modifications in OAds.

OV mutant	Genetic modifications	Modification aims
Oncorine (H101)	E1B-55K/E3 deletion	Replicated in cancer cells with aberrant p53 function and improved the safety.
DNX-2401 (Delta-24-RGD)	E1A-24-base pair deletion; RGD medication.	Selectively and efficiently replicated in cancer cells.
ICOVIR17	A 24-base pair deletion in the Rb-binding domain of E1A; insertion of E2F binding sites in the E1A promoter; the SPAM1 gene encoding PH20 hyaluronidase after the fiber.	Selectively replicated in cancer cells; modification of extracellular matrix.
Enadenotucirev	A group B Ad11p/Ad3 chimeric	High level of stability in blood and selectively replicated in cells derived from epithelial tumors.
ONYX-015 (Ad dl1520)	E1B–55-kD deletion	Selectively replicated in and destroyed tumor cells carrying mutations of the p53 tumor suppressor gene.
CG7870 (CV787)	Probasin promoter; PSA promoter; reinsertion of the E3 region.	Replicated preferentially in prostate tissue.
VCN-01	E1ACR2 deletion; E2F-binding sites insertion PH20 hyaluronidase insertion RGD insertion in the fiber knob.	Selectively replicated within tumor cells that have deregulation of the pRB, increasing tumor targeting and decreasing hepatocyte tropism.
CG7060 (CN706 and CV706)	E3 deletion; Insertion PSA promoter/enhancer.	Restricted replication primarily to cells expressing PSA, and inducing cytolysis primarily in PSA-producing cells.
OBP-301	The hTERT promoter	Improved the ability to selectively replicate in tumor cancers.
OBP-401	The hTERT promoter; GFP gene.	Selectively replicated in cancer cells.
OBP-502	RGD fiber-modified OBP-301 variant	Induced ICD and enhances the antitumor efficacy.
OBP-702	Armed tumor-suppressor p53	Induced ICD with secretion of ATP and HMGB1 in murine OS cells more strongly than OBP-301.
CRAd-S.pk7	Survivin promoter	Targeted endometriosis with a cell-killing effect.
GD55	E1B55-kD deletion; GOLPH2 promoter.	Elicited cytotoxic effects on prostate CSC-like cells.
ZD55-F-HI-sPD-1-EGFP	A PD-1 epitope (70-77aa) inserted into HI loop of fiber.	Enhanced viral infectivity and transgene delivery efficiency in PD-L1-positive tumor cells.
Ixovex-1	E1B-mutated	Significantly inhibited tumor growth.
ZD55	Conditionally replicating adenovirus type 5 with E1B (55-kDa)-deleted.	Specifically replicated and induce cytopathic effects in tumor cells.
Ad5_NULL-A20_	Genetic insertion of A20 peptide (NAVPNLRGDLQVLAQKVART) within the fiber knob protein.	Selectively targeted to αvβ6 integrin-expressing cells.
dl922-947	24-bp deletion in E1A-Conserved Region 2.	dl922-947-induced reduction of IL-8 and CCL2 production correlates with impaired tumor angiogenesis and decreased macrophage density.

RGD, arginine-glycine-aspartic; RB, retinoblastoma protein pathway; ICD, immunogenic cell death; hTERT, human telomerase reverse transcriptase; GFP, green fluorescent protein; HMGB1, high mobility group box 1; OS, overall survival. GOLPH2 (also called GP73) is a Golgi glycoprotein, which has been identified as a novel tumor marker upregulated in various cancers. CSC, cancer stem cell.

### 2.2 Activation of antitumor immune response

The therapeutic efficacy of OVs extends beyond their ability to replicate, with a robust antitumor immune response being integral to their success. Remarkably, even replication-incompetent or inactivated OVs can trigger immunogenic responses. Similar to radiotherapy and chemotherapy, OVs can induce ICD, a form of regulated cell death (RCD) that activates adaptive immunity (39). Different OVs activate distinct ICD pathways. For instance, adenoviruses primarily induce ICD through autophagy, necrosis, and pyroptosis, processes driven by the release of damage-associated molecular patterns (DAMPs), pathogen-associated molecular patterns (PAMPs), and TAAs (40, 41). DAMPs consist of intracellular signaling molecules such as calreticulin (CRT), heat shock proteins (HSPs), and high-mobility group box 1 (HMGB1), while PAMPs comprise viral nucleic acids, capsid components, and structural proteins (42–44). These immunostimulatory signals recruit dendritic cells, activate natural killer (NK) cells, and promote the infiltration of antigen-presenting cell (APC) into the TME. As a result, cytokines and chemokines are secreted, triggering inflammatory responses. DCs subsequently prime CD4^+^ and CD8^+^ T cells via MHC II/I antigen presentation, which in turn activate adaptive immune response (45).

In order to improve efficacy, engineered OVs are strategically designed to express a range of immune-modulatory factors that effectively counteract the immunosuppressive TME. ONCOS-102, a chimeric OAd encoding granulocyte-macrophage colony-stimulating factor (GM-CSF), enhances the maturation of APCs while sustaining intratumoral viral replication and promoting cellular immunity (9). Likewise, CG0070 (cretostimogene grenadenorepvec), CGTG-102, and CGTG-602 leverage GM-CSF expression to potentiate their antitumor activity (46–49). TILT-123 simultaneously expresses tumor necrosis factor-alpha (TNF-α) and interleukin-2 (IL-2), driving T cell activation while reprogramming the immunosuppressive microenvironment (50). Ad5-PC is a novel platform engineered to express a bispecific PD-1/CD137L fusion protein, promoting sustained cytotoxic T lymphocytes (CTLs) persistence and heightened immune activation, ultimately resulting in durable tumor control in hepatocellular carcinoma (HCC) (51). Multiple other immunomodulatory factors, including IL-12, CXCL10, and CD40L, have been successfully integrated into the oncolytic viral vector platform (**Table 2**).

**Table 2:** Encoding of immunostimulatory transgenes by OAds.

Name	Transgenes
ONCOS-102	GM-CSF
CG0070	GM-CSF
CGTG-102	GM-CSF
CGTG-602	GM-CSF
TILT-123	TNFα, IL-2
Ad5-PC	PD-1, CD137L
Ad-TD-nsIL12	IL-12
LOAd703	TMZ-CD40L,4-1BBL
LOAd732	Trimerized membrane-bound CD40L, 4-1BBL, IL-2
Adv-CXCL10	CXCL10
TILT-517	Full length human IL-7 sequence
CRAd-IL12-IL15	IL-12, IL-15
Ad5/3-E2F-d24-vIL2	vIL-2 protein
Delta-24-RGDOX	OX40L
MEM-288	CD40L, IFNβ
AdAPT-001	TGF-ß trap
OAd/IL12/GM-RLX	Coexpresses RLX, IL-12, GM-CSF
GD55-LHPP	LHPP gene
TILT-322	Human aMUC1aCD3 T cell engager, IL-2
TILT-452	vIL-2
Ad5sPVR	sPVR
Ad5sPD1PVR	Containing PD-1 and the PVR
LOAd713	Encoding a single chain fragment against the IL-6R in combination with a gene encoding a TMZ human CD40L.
rAd.sT	Soluble transforming growth factor receptor II fused with human IgG Fc fragment (sTGFβRIIFc) gene.
VEGF‐CRAd	Application of VEGF promoter‐based
RdB/IL12/shVEGF	IL-12, shVEGF
Ad-CCL20-CD40L	CCL20, CD40L

GM-CSF, granulocyte macrophage colony-stimulating factor; TMZ, trimerized membrane-bound isoleucine zipper; vIL-2, a human variant IL-2; sPVR, soluble extracellular domain of poliovirus receptor; RLX, relaxin; shVEGF, vascular endothelial growth factor (VEGF)-specific short hairpin ribonucleic acid.

### 2.3 Progress in clinical studies

Clinical trials investigating the use of OVs for the treatment of head and neck tumors have demonstrated significant therapeutic potential. In a Phase I trial of DNX-2401 monotherapy for patients with recurrent glioblastoma, 20% of participants survived for over three years. Notably, three patients experienced up to a 95% reduction in tumor volume, with histopathological analysis confirming substantial immune cell infiltration (52). A subsequent Phase I dose-escalation study in patients with diffuse intrinsic pontine glioma (DIPG) reported a median overall survival (mOS) of 17.8 months, with three patients surviving beyond 24 months. Remarkably, one patient with wild-type H3 and IDH1 mutations achieved sustained remission (53). Ongoing clinical trials evaluating DNX-2401 include NCT01956734 and NCT03896568, continue to explore its therapeutic potential.

In advanced HCC, monotherapy with OBP-301 has been shown to enhance CD8^+^ T cell infiltration, although tumor regression remains limited, indicating its potential for synergistic use with ICIs (54). A Phase I trial (NCT03172819) is currently assessing the efficacy of OBP-301 in combination with pembrolizumab for the treatment of advanced solid tumors. Similarly, ONCOS-102 has been demonstrated to elicit both localized and systemic CD8^+^ T cell responses, while also upregulating PD-L1 expression in a Phase I trial (9).

OAds have demonstrated significant potential not only in solid tumors but also in other malignancies. A retrospective study highlighted the efficacy of H101 in treating malignant ascites, reporting an ascites response rate (ARR) of 40% and an ascites control rate (ACR) of 75% (55). Phase II clinical trials have confirmed the safety of intraperitoneal H101 injection and revealed enhanced immune checkpoint interactions between CD8^+^ T cells and myeloid cells in long-term responders, as evidenced by CellPhoneDB analysis (56). The therapeutic effect may be attributed to the unique characteristics of the ascites microenvironment, which lacks a physical barrier, thereby facilitating viral spread. In addition, other OAds, such as enadenotucirev, AdAPT-001, and YSCH-01, have shown clinical promise in the treatment of malignant tumors (**Table 3**).

**Table 3:** The clinical trials exploring the efficacy of OAds monotherapy.

Name	Tumor type	Outcomes	Delivery	Phase	Identifier
ONCOS-102	Solid tumors refractory	Infiltration of CD8^+^ T cells.	IT	I	NCT01598129
TILT-123	Advanced solid cancers	TILT-123 was safe and able to produce antitumor effects in local and distant lesions in heavily pre-treated patients.	IV, IT	I	NCT04695327
DNX-2401 (Delta24-RGD)	Recurrent malignant glioma	Tumor infiltration by CD8^+^ T and T-bet^+^ cells, and transmembrane immunoglobulin mucin-3 downregulation after treatment.	IT	I	NCT00805376
	DIPG	OS 17.8 months, 3 of whom lived longer than 24 months.	IT	I/II	NCT03178032
OBP-301	Liver cancer	MTD 6×10^12^ viral particles, infiltration CD8^+^ T cell.	IT	I	NCT02293850
H101 (Oncorine)	MA	Increased tumor cell lysis and tumor-specific CD8^+^ T cells were identified, achieving an ascites control rate of 75%.	IP	II	NCT04771676
Enadenotucirev	Epithelial solid tumors	MTD 3 × 10^12^ viral particles.	IV	I	NCT02028442
	CRC, NSCLC, UCC, RCC	Both IV and IT injection are feasibility and good tolerability. Treatment-related adverse effects were more common but less severe after IV injection.	IT, IV	I	NCT02053220
NSC-CRAd-S-pk7	Malignant glioma	PFS 9.05 months, OS 18.4 months.	IT	I	NCT03072134
YSCH-01	Advanced solid tumors	ORR was 27.3%, DCR was 81.8%. mPFS was 4.97 months, mOS was 8.62 months.	IT	I	NCT05180851
AdAPT-001	Advanced refractory solid tumors	Demonstrated an acceptable safety and tolerability profile, with 20% of patients in partial remission and more than 30% of patients prolonging stabilization for ≥6 months.	IT	I	NCT04673942

DIPG, diffuse intrinsic pontine glioma; CRC, colorectal cancer; NSCLC, non-small-cell lung cancer; UCC, urothelial cell cancer; RCC, renal cell cancer; IT, intratumoral injection; IV, intravenous injection; IP, intraperitoneal injection; MTD, maximum tolerated dose; mPFS, median progression-free survival; mOS, median overall survival; ORR, overall response rate; DCR, disease control rate; MA, malignant ascites.

Despite their potential, the clinical application of OVs in treating “cold tumors”, which are characterized by limited immune infiltration, faces several challenges. These include uncertainties surrounding long-term efficacy and the impact of tumor heterogeneity on the success of monotherapy treatments.

### 2.4 Safety

Wild-type adenoviruses often cause off-target infections in normal tissues due to their lack of tumor specificity. In contrast, engineered OAds exhibit improved tumor selectivity through various genetic modifications. For example, ONYX-015 enables tumor-specific replication in p53-deficient cells through a deletion of the E1B55K gene, while H101 enhances selectivity by introducing double deletions of the E3 and E1B55K genes. The ZD55 platform combines the E1B55K deletion with a transgene insertion site, creating a bifunctional design (32, 57, 58). Moreover, OVs such as CG7870, CG7060, OBP-301/401, GD55, and CRAd-S.pk7 restrict viral replication to tumor cells by utilizing tissue-specific promoters, including prostate-specific antigen (PSA) and human telomerase (59–65).

In addition to optimizing targeting, viral tropism plays a crucial role in OAd therapy. While natural adenoviruses primarily infect CAR receptor-positive cells through fibronectin binding, genetically engineered OAds are capable of precisely targeting CAR-negative tumor cells, thereby reducing the risk of infecting normal tissues. Specifically, serotype 5 human adenovirus exhibits a strong hepatic tropism when administered intravenously, as it is captured by Kupffer cells and primarily localizes to hepatocytes. This pronounced hepatic targeting poses a significant challenge for clinical application (66, 67). Surface charge modifications, such as the HAdV-5-HexPos3_ΔCAR variant, have shown substantial potential in reducing non-target organ tropism (67).

Genetically modified OVs typically exhibit a favorable safety profile, with a low incidence of severe adverse events or the need for therapy discontinuation. Common side effects include transient systemic symptoms such as fever and fatigue, as well as localized inflammation at the injection site. However, a therapeutic paradox remains: achieving optimal intratumoral viral titers while maintaining systemic safety is a significant challenge due to the dose-dependent nature of the clinical response.

### 2.5 Delivery methods and immunological clearance

OVs are predominantly delivered through two routes: IT injection and IV injection. Alternative methods, such as intraperitoneal (IP) injection, subcutaneous (SC) injection, and intravesical (IVE) injection, are less commonly used (68–70). In preclinical studies, the choice of delivery route is typically determined by the specific experimental objectives, whereas in clinical practice, patient safety is the foremost consideration. IT injection is the standard method for treating accessible tumors, though it has limitations when targeting deep or metastatic lesions (71). Additionally, IT injection carries risks, including bleeding, infection, and tumor seeding, which require specialized clinical expertise to manage effectively.

IV administration offers systemic delivery, thereby overcoming the limitations of IT injection and enhancing clinical feasibility. Nevertheless, pre-existing antiviral antibodies and serum protein binding can significantly reduce the bioavailability of the virus (72). Hepatic sequestration by Kupffer cells poses another significant challenge, as these macrophages rapidly clear circulating adenoviral particles, thereby limiting the effective delivery of therapeutic payloads (73, 74). To address these challenges, researchers have developed several strategies, including capsid modifications, liposomal formulations, cell-based delivery systems (e.g., neural stem cells, mesenchymal stem cells, T cells, tumor-infiltrating lymphocytes [TILs], NK cells, DCs, and human dental pulp stem cells), extracellular vesicles, gelatin hydrogels, and nanomaterials (65, 75–85).

The immunogenicity of OVs plays a critical role in determining the appropriate route of administration. Highly immunogenic variants are better suited for localized IT delivery, while strains with lower immunogenicity may be more effective for prolonged systemic circulation through IV administration (86). Additionally, it is essential to consider the interaction between delivery platforms and the tumor immune microenvironment to mitigate potential immunosuppressive effects. Developing advanced delivery systems that enhance viral bioavailability while ensuring a strong safety profile is critical for achieving therapeutic concentrations at the tumor site.

## 3 Other OVs under investigation

### 3.1 Herpes simplex virus

Herpes Simplex Virus (HSV) is an enveloped double-stranded DNA virus that possesses both a nucleocapsid and an outer membrane (87). It exists in two serotypes: HSV-1 and HSV-2. HSV-1 holds considerable antitumor potential due to its large genome, which can accommodate multiple exogenous genes, as well as its ability to evade immune responses (88). Engineered HSV-1-based oncolytic viruses, such as Talimogene laherparepvec (T-VEC) and G47Δ, have been approved for the treatment of malignant melanoma and gliomas, respectively (89, 90).

### 3.2 Vaccinia virus

Vaccinia virus (VV) is a double-stranded DNA virus of approximately 190kb in size, belonging to the poxvirus family (91). Known for its excellent safety profile and rapid replication cycle, VV has emerged as a leading platform for oncolytic virotherapy (92, 93). Genetically modified strains, such as JX-594 and Pexa-Vec, are currently undergoing clinical trials. Notably, JX-594 has shown promising results for IV administration and demonstrated resistance to neutralization by antibodies and complement (94, 95).

### 3.3 Reovirus

Reovirus (RV) is a non-enveloped double-stranded RNA virus that primarily causes mild upper respiratory or gastrointestinal infections (96, 97). Pelareorep, a type 3 reovirus, has shown suitability for IV administration and boasts an excellent safety profile. It has demonstrated antitumor efficacy in several clinical trials, positioning it as one of the most advanced oncolytic RNA virus therapeutics (98).

### 3.4 Measles virus

Measles virus (MeV) is an enveloped, single-stranded negative-sense RNA virus that naturally targets tumors (99). Its excellent safety profile and the absence of dose-limiting toxicity make it a promising candidate for oncolytic virotherapy (100).

### 3.5 Newcastle disease virus

Newcastle disease virus (NDV) is an enveloped, single-stranded negative-sense RNA virus with a substantial capacity for exogenous gene insertion. Its P/M gene intergenic region serves as an ideal site for genetic modification (101, 102). Early clinical trials using the wild-type NDV strain have demonstrated good patient tolerance (103).

Other viruses, including coxsackievirus, seneca valley virus, poliovirus, and vesicular stomatitis virus (VSV), have also shown promise in antitumor therapy (104–107). Each of these viruses possesses unique biological properties, providing diverse therapeutic options for tumor treatment and broadening the potential applications of oncolytic virotherapy.

## 4 Immune checkpoint inhibitors

### 4.1 Mechanisms

#### 4.1.1 PD-1/PD-L1 and CTLA-4/B7 pathways

ICIs play a pivotal role in enabling tumors to escape immune surveillance. By blocking these inhibitory pathways, ICIs enhance T cell responses targeting tumor cells. Cytotoxic T lymphocyte-associated protein 4 (CTLA-4), a homolog of the co-stimulatory receptor CD28, negatively regulates T cell activation. While CD28 promotes T cell activation by binding B7 molecules (CD80/CD86) on DCs, CTLA-4 competes with CD28 for binding to B7 with a higher affinity, thereby inhibiting T cell activation (108, 109). Furthermore, inhibition of CTLA-4 disrupts the function of Tregs, which inherently express high levels of CTLA-4 and are essential in mediating immune suppression within the TME (110, 111).

PD-1, a key member of the CD28 superfamily, interacts with two ligands: PD-L1 (B7-H1) and PD-L2 (B7-DC) (112, 113). Upon binding, PD-1 triggers inhibitory signaling by recruiting SHP-2, a phosphatase that dephosphorylates critical signaling molecules in the T cell receptor (TCR) pathway, including ZAP-70 and CD3ζ (114–116). This cascade of signaling events impairs T cell activation, proliferation, and cytokine production, thereby diminishing the antitumor immune response. As second-generation ICIs, PD-1/PD-L1 inhibitor have shown clinical efficacy and are approved for various cancers, including melanoma, NSCLC, and renal cell carcinoma (RCC). However, despite enhancing T cell activation, targeting CTLA-4 or PD-1 alone is insufficient to fully control tumor progression.

#### 4.1.2 Novel immune checkpoint inhibitors

To enhance therapeutic outcomes, research has increasingly focused on identifying additional immune checkpoint targets. Novel co-inhibitory receptors currently under investigation include lymphocyte activation gene-3 (LAG-3), T cell immunoreceptor with immunoglobulin and ITIM domain (TIGIT), T cell immunoglobulin and mucin-domain containing-3 (TIM-3), and signal regulatory protein alpha (SIRPα). Concurrently, co-stimulatory receptors such as inducible T cell costimulator (ICOS), members of the TNF receptor superfamily (e.g., OX40 and 4-1BB), and Toll-like receptors (TLRs) have emerged as promising candidates for therapeutic targeting.

LAG-3, also known as CD223, is a transmembrane glycoprotein structurally similar to CD4 (117). The first FDA-approved LAG-3 inhibitor, Opdualag, combines the anti-LAG-3 antibody relatlimab-rmbw with the anti-PD-1 antibody nivolumab. Approved in 2022, Opdualag is used for the treatment advanced melanoma treatment (118).

TIGIT, predominantly expressed on T cells, NK cells, Tregs, and tumor-infiltrating lymphocytes (TILs), binds to the ligands CD155 and CD112 (119, 120). By competing with CD226 for binding to CD155, TIGIT suppresses NK cell-mediated cytotoxicity. Blocking both TIGIT and PD-1 can restore CD226 signaling and enhance CD8^+^ T cell responses (121–123). Clinical trials with agents such as tiragolumab, vibostolimab, and tislelizumab have demonstrated promising results.

TIM-3 is expressed on exhausted T cells and interacts with several ligands, including phosphatidylserine and galectin-9, to promote immune tolerance (124). The binding of TIM-3 to MHC II directly suppresses T cell proliferation. While preclinical studies have demonstrated therapeutic potential, a bispecific antibody targeting TIM-3 and PD-L1, known as LY3415244, exhibited unexpected immunogenicity, leading to the discontinuation of clinical trials (124–126).

SIRPα binds to CD47, sending a “don’t eat me” signal to macrophages, which inhibits phagocytosis (127, 128). Blocking the CD47-SIRPα axis can promote tumor clearance by macrophages.

#### 4.1.3 Novel immune checkpoint stimulators

ICOS, a member of the CD28 family, is expressed on activated T cells and enhances T cell responses by binding to ICOS-L and activating the PI3K signaling pathway (129). Preclinical studies have demonstrated that blockade of CTLA-4 upregulates ICOS on CD4^+^ T cells, prompting the ongoing Phase II clinical trial of the ICOS agonist vopratelimab in combination with ipilimumab for PD-1/PD-L1-resistant NSCLC (NCT03989362).

The 4-1BB receptor (CD137), which is expressed on activated T and NK cells, has emerged as a promising target for treating solid malignancies (130, 131). However, systemic administration of 4-1BB agonists has been associated with dose-limiting hepatotoxicity (132, 133). TLRs within TME promote tumor progression by activating the NF-κB pathway and inducing immunosuppressive cytokines. In contrast, TLR7/8 agonists have been shown to enhance antitumor immunity when used in combination with immune checkpoint blockade (ICB) (134, 135).

ICB has become a key therapeutic strategy for advanced malignancies, with current research focusing on three main objectives: improving clinical outcomes by enhancing efficacy and reducing irAEs, developing predictive biomarkers for stratify treatment responses, and advancing combinatorial therapeutic approaches. Despite these advancements, some translational barriers remain, particularly in maintaining immunological equilibrium and addressing intratumoral heterogeneity.

### 4.2 Resistance to treatment with ICIs

The introduction of ICIs has significantly transformed the treatment landscape for various cancer types; however, ongoing challenges such as treatment resistance and the optimization of therapeutic protocols remain critical areas for research. Resistance to ICIs can be categorized into two main types: primary resistance, which is characterized by the absence of an initial therapeutic response due to inherent tumor characteristic, and acquired resistance, which occurs after an initial clinical response followed by disease progression (4).

Mechanistically, resistance to ICIs arises from both tumor-intrinsic and microenvironmental factors. Intrinsic mechanisms encompass a low burden of neoantigens, dysregulation of oncogenic signaling and metabolic pathways, impaired type I interferon (IFN-I) signaling, defective antigen processing and presentation, and epigenetic modifications (136). Extrinsic mechanisms involve insufficient T cell infiltration, the expansion of immunosuppressive cell populations (such as myeloid-derived suppressor cells [MDSCs] and Tregs), the induction of alternative immune checkpoints (e.g., TIM-3, LAG-3), epithelial-mesenchymal transition (EMT), pro-angiogenic signaling, and dysbiosis of the gut microbiome (137).

Tumors are commonly categorized as immunologically “hot” (inflamed) or “cold” (non-inflamed) based on TME characteristics, including profiles of inflammatory cytokines and the infiltration of CD8^+^ T cells. Primary resistance is typically observed in “cold” tumors, which exhibit minimal immune cell infiltration, while acquired resistance often develops in initially responsive “hot” tumors through adaptive immune-editing processes (138–140). This classification has clinical significance, as “cold” tumors typically demonstrate low PD-L1 expression and poor responses to immunotherapy. A key mechanism of resistance is T cell exhaustion, which results from prolonged antigen exposure. Exhausted T cells are characterized by: 1) upregulation of co-inhibitory receptors (PD-1, CTLA-4, LAG-3), 2) metabolic dysfunction, and 3) reduced secretion of effector cytokines (IFN-γ, TNF-α, IL-2) (141). Although PD-1 blockade can temporarily restore T cell functionality through epigenetic modifications, complete recovery is often not achieved, as terminally exhausted T cell clones persist even after ICI therapy.

IFN-γ illustrates the dual role of immune regulation, as it promotes antitumor immunity by upregulating MHC I upregulation while simultaneously facilitating immune escape through the expression of PD-L1 and the recruitment of regulatory T cells (Tregs) (142, 143). Current therapeutic strategies are increasingly focused on rationally designed combination therapies, with promising synergistic effects observed between ICIs and OAds. OAds enhance the immune response by reconfiguring immunosuppressive networks within the TME.

## 5 Combination strategy reverses drug resistance

### 5.1 Combination therapy optimizes antitumor efficacy

**Figure 1:**
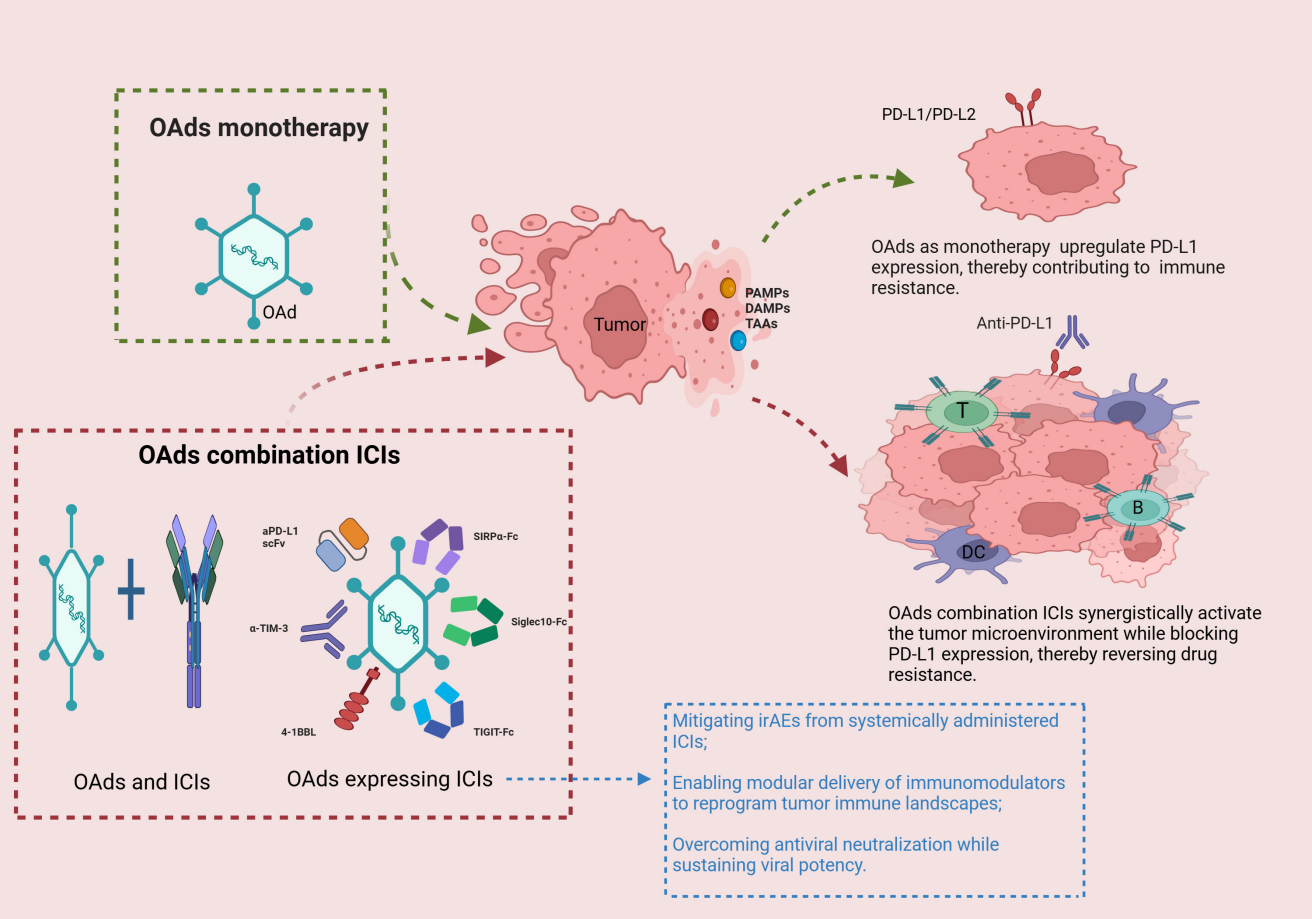
Synergy of oncolytic adenovirus (OAds) and immune checkpoint inhibitors (ICIs). OVs lyse tumor cells and induce immunogenic cell death (ICD) in tumor cells, releasing damage-associated molecular patterns (DAMPs), pathogen-associated molecular patterns (PAMPs) and soluble tumor-associated antigens (TAAs), which stimulate innate and adaptive immune responses, as well as inducing upregulation of PD-L1 expression in tumor cells, leading to T cell exhaustion. However, PD-L1 expression can be blocked by OAds in combination with ICIs (mainly OAds and ICIs, OAds expressing ICIs), thereby reversing resistance. In addition, OVs expressing ICIs have the following advantages over separate individual dosing: reducing immune-related adverse events (irAEs) associated with systemic ICI therapy; flexible delivery of cytokines, immune checkpoint molecules, and other immunomodulators to further activate the immune microenvironment; diminishing virus-induced neutralization and ensuring effective viral load. Created with BioRender.com.

Current methodologies for the integration of OVs and ICIs are primarily categorized into two distinct strategies: 1) transgene delivery systems using engineered oncolytic adenoviral vectors (OAds expressing ICIs), and 2) spatiotemporally separated delivery of OAds and ICIs (OAds and ICIs). The first strategy presents several advantages compared to conventional approaches, and certain mechanisms underlying this combination therapy are depicted in **Figure 1**. we conducted a search of the PubMed database for promising preclinical and clinical studies examining the integration of OVs and ICIs over the past five years, as summarized in **Table 4**.

**Table 4:** Preclinical and clinical trials combining OAds with ICIs (2019-2024).

Combination	Name	Combination agents	Cancer type	Delivery	Outcomes	Phase	Reference
OAds and ICIs	OBP-502	Anti-PD-1	Colon cancer Pancreatic cancer	IT	Recruited CD8^+^ T cells	Pre-	(144)
	XVir-N-31	Nivolumab	GBM	IT	Abscopal Effects	Pre-	(13)
	OBP-702	PD-L1 blockade	Pancreatic cancer	IT, IP	Suppressed GM-CSF-mediated MDSC accumulation.	Pre-	(138)
	Adv-CXCL10	Anti-PD-1	Colon cancer	IT	Increased the number of CXCR3^+^ T cells in the TME.	Pre-	(146)
	TILT-123	Anti-PD-1	NSCLC	IV	Decreased percentage of ITAMs and improved DC cell maturation.	Pre-	(148)
		Anti-PD-1/anti-PD-L1	OC	IP	Induced T cell activation, caused positive microenvironment changes.	Pre-	(14)
		Anti-PD-1/anti-PD-L1	Refractory Head and Neck Cancer	IT	Induced tertiary lymphoid structure formation.	Pre-	(140)
		Anti-PD-L1	PDAC	IT	Improved tumor growth control further and demonstrated good safety and toxicity profiles.	Pre-	(151)
		Anti-PD-L1	Urological tumor	IT	Increased T-cell trafficking signals.	Pre-	(149)
		Anti-PD-1	Melanoma	IT	Increased the CD8^+^/CD4^+^ T cell ratio.	Pre-	(147)
	ZD55-IL-24	Anti-PD-1	Melanoma	IT	Promotion of tumor immune infiltration.	Pre-	(152)
	rAd.GM	Anti-PD-L1, anti-CTLA-4	TNBC	IT	Inhibited tumor growth and prolonged survival; recruited CD8^+^ T and T memory cells, promoted M1 phenotype, reduced Tregs and TAMs.	Pre-	(12)
	Enadenotucirev	Nivolumab	Epithelial cancer	IV	mOS 16.0 months,69% were alive at 12 months; increase in intra-tumoral CD8^+^ T cell infiltration.	phase 1	(38)
	H101	Nivolumab	HCC	IT	ORR 11.1%; reversed immunological resistance.	Phase 1	(15)
	DNX-2401 (Delta24-RGD)	Anti-PD-1	GBM	IT	Induced long-term survival with PD-1 blockade.	Pre-	(153)
		Pembrolizumab	GBM	IT	OS at 12 months was 52.7% (95% CI 40.1–69.2%)	Phase 1/2	(154)
	CG0070	Nivolumab	MIBC	IVE	Pathologic complete response rate of 42.1%	Phase 1	(49)
		Pembrolizumab	Bladder cancer	IVE	12- month CR 57.1% 24-month CR 51.4%	Phase 2	(155)
OAds expressing ICIs	Ad-Cab	IgGA Fc PD-L1	Various cancers	IT	Resulting in neutrophil activation	Pre-	(156)
	RCAd-LTH-shPD-L1	Armed with a DNA fragment encoding an anti-VEGF antibody	Mucinous gastric carcinoma Astroglioma	IP	Inhibited PD-L1 expression, upregulated the secretion of IFN-γ, IL-6, and IL-12, and increased the proportions of CD4^+^ T and CD8^+^ T cells.	Pre-	(157)
	CAV2-AU-M2	Anti-PD-1 sdAb	OS	IT	Facilitated the secretion of anti-PD-1 sdAb locally in the TME and therefore prevented adverse effects.	Pre-	(159)
	ZD55	aPD-L1 scFv	TNBC	IT	ZD55-aPD-L1 scFv is superior to co-administration of ZD55 and systemic anti-PD-L1 antibody.	Pre-	(158)
	Ad-GD55-α-TIM-3	α-TIM-3	HCC	IT	Inhibited tumor growth and engaged in a more robust local immune response.	Pre-	(160)
	Delta-24-ACT	4-1BBL	DIPG	IT	Increased the number and improved the functionality of immune cells.	Pre-	(130)
	SG635-SF	Signal regulatory protein‐α (SIRPα) ‐IgG1 Fc fusion gene	OC	IT	Antitumor effect of SG635‐SF was CD47‐dependent.	Pre-	(161)
	ONCOS-204	ICOSL	EGFR^+^ tumor cells	IT	Increased CD4^ + ^T cells; Enhanced functional activity of tumor-specific BsAbs.	Pre-	(162)
	VALO-D102	CD40, OX40L	Melanoma	IT	Increased tumor-specific T cell responses, reduced tumor growth, and induced systemic anti-cancer immunity.	Pre-	(163)
	OAd-null	SIRPα-Fc Siglec10-Fc TIGIT-Fc	Breast cancer Colon cancer Glioma cell Lung carcinoma	IT	OAd-SIRPα-Fc,OAd-Siglec10-Fc Macrophage cell; OAd-TIGIT-Fc CD8^+^T cell.	Pre-	(18)
	AdV5/3-D24-ICOSL-CD40L	Pembrolizumab Paclitaxel	Breast cancer	IT	Reduced the tumor volume; increased infiltration of CD8^+^ T, CD4^+^ T and Tregs cells.	Pre-	(164)
		Mesothelioma	Mesothelioma	IT	Improved anticancer efficacy and survival by targeted cancer cell destruction and triggered of ICD.	Pre-	(165)
	mLOAd703	Anti-PD-1 Anti-PD-L1 Anti-TIM-3	Melanoma	IT, IP	Reduced tumor growth; Abscopal responses.	Pre-	(166)

IT, intratumoral Injection; IP, intraperitoneal perfusion; IVE, intravesical injection; ITAMs, immunosuppressive tumor-associated macrophages; pre-, preclinical trial; NSCLC, non-small-cell lung cancer; PDAC, pancreatic ductal adenocarcinoma; TNBC, triple negative breast cancer; HCC, hepatocellular carcinoma; DIPG, diffuse intrinsic pontine gliomas; GBM, glioblastoma; OC, ovarian cancer; MIBC, muscle invasive bladder cancer; OS, osteosarcoma; scFv, Single-chain variable fragment; sdAb, Single-domain antibody; ICD, Immunogenic cell death; SIRPα,Signal regulatory protein alpha; TIGIT, T cell immunoreceptor with immunoglobulin and ITIM domain; CR, Complete remission.

### 5.2 Progress in preclinical and clinical research

#### 5.2.1 OAds and ICIs

Initial validation of the effectiveness of combining the two immunotherapeutic agents has been carried out using various preclinical animal models. OBP-301 is a novel attenuated type 5 adenovirus that utilizes the hTERT promoter to enhance the expression of the adenovirus early in areas linked to an internal ribosome entry site (IRES) sequence. This genetic construct facilitates tumor-specific viral replication and induces lytic cell death in a variety of cancer cell types (54). Its derivative, OBP-502, has been shown to induce ICD in models of CRC and pancreatic ductal adenocarcinoma (PDAC), functioning synergistically with PD-1 blockade to promote CD8^+^ T cell infiltration and enhance systemic antitumor immunity (144). Additionally, the gemcitabine-resistant variant OBP-702 has been found to counteract GM-CSF-mediated immunosuppression, thereby further improving the efficacy of PD-L1 blockade in PDAC (138). Engineered oncolytic vectors facilitate multimodal immunomodulation through various mechanisms. The combination of ONCOS-102 with pembrolizumab has been shown to significantly reduce tumor burden, while cytokine-armed viral constructs effectively reprogram the immunosuppressive TME (145). Adv-CXCL10, a recombinant adenovirus encoding CXCL10, enhances the effectiveness of PD-1 inhibitors by promoting the expansion of CXCR3^+^ T cells (146). Furthermore, the bifunctional agent TILT-123, which expresses TNFα and IL-2, alters the immune landscape by the ratios of CD8^+^ to CD4^+^ T cells and promoting the maturation of DC across various cancer models, including the induction of tertiary lymphoid structures in head and neck cancers (14, 140, 147–151). Similarly, ZD55-IL24 has been shown to mitigate immune exclusion in non-inflamed tumors (152). The use of multi-agent combinations may further enhance therapeutic effectiveness. In a model of triple-negative breast cancer (TNBC), OAds demonstrated a synergistic effect when combined with anti-PD-L1 and anti-CTLA-4 immunotherapies, resulting in improved tumor control and extended survival, with 20% of the subjects exhibiting complete suppression of metastasis (12).

Clinical trials provide additional evidence supporting the potential of integrating the two approaches to facilitate clinical translation. Substantial advancements have been achieved in the research pertaining to cancer of the gastrointestinal tumors. In a Phase I clinical trial, the administration of enadenotucirev as a monotherapy resulted in the infiltration of CD8^+^ T cells within microsatellite stable (MSS) colorectal cancer (CRC) tumors, indicating the potential for virus-induced immunogenicity (10). A subsequent investigation that combined enadenotucirev with nivolumab demonstrated improvements in OS and T cell activation in MSS CRC patients (38). In patients with refractory HCC, the combination of H101 and nivolumab yielded an objective response rate (ORR) of 11.1% and a mOS of 15.04 months (15). Importantly, certain individuals classified as having stable disease (SD) experienced extended OS with ongoing treatment, highlighting the need for further investigation of combination therapies in larger clinical trials.

In the treatment of head and neck tumors, DNX-2401 has shown efficacy in reversing PD-1-mediated T cell exhaustion, leading to sustained remission in patients with glioma (153). A Phase 1/2 clinical trial indicated a 12-month overall survival rate of 52.7% for IDH1 wild-type gliomas, thereby reinforcing the potential of this therapeutic strategy for treating refractory tumor types (154). In the context of urological tumors, the Phase 2 CORE-001 trial demonstrated that the combination of CG0070 and pembrolizumab in patients with Bacillus Calmette-Guérin (BCG)-unresponsive bladder cancer yielded favorable risk-benefit profiles, with a complete remission (CR) rate of 57.1% at 12 months and 51.4% at 24 months (155). Additionally, ongoing prospective trials are assessing the efficacy of H101 in conjunction with PD-1 inhibitors for patients with advanced malignant pleural mesothelioma or non-muscle invasive bladder cancer who have not responded to previous treatments (NCT06031636, NCT05564897). Furthermore, investigations into triple-drug combinations involving OVs, immunotherapies, and anti-angiogenic agents are currently in progress (NCT05303090).

#### 5.2.2 OAds expressing ICIs

The existence of a functional Fc region can be advantageous yet problematic, as immune checkpoints are extensively distributed throughout the organism, leading to irAEs following the systemic administration of antibodies. To mitigate this issue, Hamdan et al. developed an Fc-fusion peptide aimed at PD-L1, which included a chimeric constant region composed of IgG1 and IgA1 (IgGA) (156). This construct was subsequently incorporated into an OAd (Ad-Cab), thereby facilitating the activation of neutrophil effector functions mediated by both IgG1 and IgA1 (156). Moreover, RCAd-LTH-shPD-L1, a dual-transgene OAd, facilitates the localized administration of anti-VEGF antibodies and PD-L1-targeting short hairpin RNA (shRNA), thereby enhancing the secretion of IFN-γ, interleukin-6 (IL-6), and interleukin-12 (IL-12), while also promoting T cell infiltration (157). This strategy contributes to the normalization of tumor vasculature and the reprogramming of immunosuppressive TME networks. The anti-PD-L1 scFv-expressing OAd, ZD55-apd-L1-scFv, demonstrated superior antitumor efficacy compared to both the parental ZD55 virus and systemic anti-PD-L1 therapies (158). CAV2-AU-M2, an anti-PD-1 single-domain antibody (sdAb)-armed OAd, synergistically integrates multiple immunotherapeutic approaches to address the challenges associated with osteosarcoma treatment (159). Furthermore, the bispecific CD137 agonist/PD-L1 blocker Ad5-PC enhances CTL activity through the simultaneous activation of the CD137 pathway and blockade of the PD-1/PD-L1 interaction (51). Several combination trials utilizing novel checkpoint inhibitors have yielded encouraging results. OAds expressing TIM-3, 4-1BBL, (SIRPα)-IgG1 Fc, ICOSL, CD40L, and OX40L demonstrated antitumor effects across various preclinical tumor models (130, 160–163). This emerging strategy capitalizes on the characteristic immune cell infiltration within the TME to create recombinant OAds for targeted therapy. This approach encompasses three distinct types of recombinant OAds: OAd-SIRPα-Fc, OAd-Siglec10-Fc, and OAd-TIGIT-Fc. OAd-SIRPα-Fc and OAd-Siglec10-Fc have been shown to significantly suppress tumor growth in macrophage-rich tumor microenvironments, while OAd-TIGIT-Fc primarily enhances T cell activation (18). The targeted methodology facilitates tumor-selective immunotherapy. Other preclinical and clinical evidence supports the combination of OAd-encoded ICIs with systemic checkpoint blockade (164–166).

The localized production of antibodies by OVs enhances the specificity of therapeutic interventions while reducing the adverse effects typically associated with ICIs alone (21). Furthermore, ICIs can regulate the balance of the immune response, thereby reducing the clearance of the virus. In contrast, the independent administration of OVs and ICIs offers greater flexibility but may elevate the potential for toxicity. Therefore, the selection of the most appropriate treatment regimen should be tailored to the particular clinical context.

## 6 The integration of OAds with other treatment modalities

Beyond combination with ICIs, OAds also have demonstrated synergistic therapeutic effects when integrated with chemotherapy, radiotherapy, adoptive cell therapy (ACT), targeted therapy and other treatment modalities.

Chemotherapeutic agents have the potential to augment the effectiveness of OV therapy by attenuating the host’s antiviral immune response. For instance, the adenovirus Ad5/3-pCDX2, which is regulated by the CDX2 promoter, when administered in conjunction with 5-fluorouracil (5-FU), leads to an upregulation of CDX2 expression in tumors, thereby significantly inhibiting the proliferation of CDX2-negative CRC (167). Clinical evidence has also indicated that the combination of LOAd703 with standard chemotherapy demonstrates favorable safety profiles and preliminary antitumor activity in patients diagnosed with advanced PDAC (168). Similarly, the integration of ONCOS-102 with pemetrexed and platinum-based chemotherapeutics in the treatment of malignant pleural mesothelioma has resulted in an extended mOS of 20.3 months, markedly enhancing patient outcomes compared to chemotherapy alone, while also facilitating T cell infiltration and the expression of pro-inflammatory cytokines (169). Additional combination therapies, such as DNX2401 with temozolomide (NCT01956734) and H101 with FOLFOX (NCT05124002), are currently under investigation in clinical trials.

The integration of OVs with radiotherapy has demonstrated a distinctive synergistic effect. The release of antigens induced by radiation enhances the immune response initiated by OVs, while the viral infection simultaneously impairs the tumor cells’ capacity to repair DNA damage caused by radiation. Research has indicated that adenoviral proteins can directly disrupt the DNA damage response (DDR) pathway, thereby modulating critical processes involved in the recognition and repair of DNA damage (170). Furthermore, the recently developed PEG-coated intravenous RadioOnco formulation has effectively inhibited DNA damage repair mechanisms, resulting in the activation of durable antitumor immune responses and offering substantial benefits in the management of tumor metastasis and recurrence (171).

The synergistic effects of OVs and targeted therapy primarily rely on the precise regulation of critical signaling pathways. The JAK-STAT pathway is particularly significant in determining tumor sensitivity to OAds, as impairments within this pathway markedly increase viral susceptibility (172). JAK inhibitors, such as ruxolitinib, have demonstrated potential in augmenting the effectiveness of OV therapies; however, the outcomes are contingent upon the specific experimental models employed. For instance, in the context of VSV-IFNβ treatment, ruxolitinib was found to enhance viral activity in resistant cell lines, yet it did not yield a substantial improvement in survival rates within immune-competent models of NSCLC (173, 174). In the case of CRC, the combination of OVs with PI3K-γ inhibitors may address ICI resistance associated with the PI3K/AKT/mTOR signaling pathway. Nevertheless, challenges persist due to the potential for cross-resistance arising from compensatory mechanisms within tumor signaling pathways and the remodeling of the TME (175).

ACT, which incorporates the use of effector cells such as CAR-T cells, NK cells, DCs, or TILs, has encountered several obstacles in the treatment of solid tumors. These challenges include inadequate tumor infiltration, the presence of immunosuppressive microenvironments, T cell exhaustion, and limited cell persistence (176). The integration of genetically modified OVs with ACT has shown potential in enhancing the efficacy of CAR-T cells. For instance, CAR-T cells infected with the TS-2021 virus exhibit sustained activity through autocrine interleukin-15 (IL-15), thereby overcoming resistance in glioblastoma therapy (177). OAds that express specific chemokines can facilitate the infiltration of CAR-T cells into tumors and modify the immune microenvironment (178). Furthermore, the combination of OVs with NK cells or DCs has demonstrated notable synergistic antitumor effects (179, 180).

Innovative approaches, including photodynamic immunotherapy and high-dose vitamin C, exhibit potential for future integration with OV (181, 182). While combination therapies present considerable benefits, additional research is essential to refine safety evaluations, dosing protocols, and other relevant factors. Among these, the integration of ICIs with OVs is regarded as one of the most promising clinical strategies, supported by numerous trials that confirm its therapeutic efficacy.

## 7 Clinical challenges and future directions

While the integration of OVs and ICIs demonstrates promise for antitumor treatment, several challenges remain to be resolved. Key considerations include establishing the optimal timing for administration, achieving a balance between the antiviral and antitumor immune responses, and identifying effective predictive tumor biomarkers. Furthermore, the interplay between OVs and microbiome is currently under investigation, which may offer insights for the advancement of novel therapeutic strategies.

### 7.1 Appropriate timing for treatment

The integration of OVs and ICIs necessitates meticulous timing, especially in the synchronization of various administration approaches, including alternating, sequential, or concurrent delivery methods. Nguyen et al. established a classification framework that delineates five distinct paradigms of administration: (i) anti-PD-1 priming→OV; (ii)concurrent administration; (iii)OV priming→anti-PD-1;(iv) concurrent therapy priming→anti-PD-1; and (v)OV priming→concurrent therapy (183). Preclinical findings indicate that optimal synergy is attained when OV priming is succeeded by simultaneous dual therapy. From a mechanistic perspective, the initial administration of the OV facilitates the recruitment of TILs and creates an inflamed TME. Nevertheless, the compensatory upregulation of PD-L1 on tumor cells may diminish this therapeutic effect. The occurrence of adaptive resistance underscores the importance of implementing a sequential approach to the administration of OV in conjunction with ICI. The inhibition of PD-1 serves to alleviate T-cell exhaustion while simultaneously augmenting the antitumor immune response elicited by OV, thus preserving the integrity of the cancer-immunity cycle. In the HaP-T1 PDAC models, the “OV priming→concurrent therapy” sequence achieved pathologic complete remission, whereas the sequence of “anti-PD-1→OV” sequencing only enhanced tumor control (69). Tumor-free survivors exhibited durable immune memory, indicating the possibility of neoadjuvant applications through preoperative intratumoral delivery of OV.

The clinical development of T-VEC, a HSV engineered for tumor-selective replication, exemplifies both the potential and the challenges associated with combination immunotherapy. A Phase II clinical trial demonstrated that the combination of T-VEC with ipilimumab resulted in a higher ORR compared to ipilimumab administered alone (odds ratio, 2.9, P =0.002), while maintaining a similar safety profile (184). Conversely, a Phase III clinical trial did not reveal a significant advantage in PFS or OS when T-VEC was combined with pembrolizumab, as opposed to pembrolizumab monotherapy (185). In both studies, OV was initially employed to elicit an early anti-cancer immune response, followed by the introduction of ICIs to enhance the immune effect synergistically. However, the differing outcomes may be attributed to insufficient time in the Phase III study to fully activate the TME. Given that factors such as tumor biology, treatment design, and pharmacological parameters influence therapeutic responses, the timing of intervention is crucial for achieving efficacy and must be meticulously calibrated across various cancer types. The intricate relationship between T cell activation and the heterogeneous TME presents potential risks, including the potential for premature or excessive immunostimulation, which can lead to irAEs and accelerated T cell exhaustion.

### 7.2 The balance between the antitumor response and antiviral response

Enhancing the efficacy of OV therapy necessitates a careful equilibrium between the antitumor response and the host’s antiviral immune response. This issue is notably exemplified by wild-type adenoviruses, which face obstacles posed by pre-existing neutralizing antibodies that impede their spread (72). Tumor-intrinsic defense mechanisms, such as the phosphorylation of PKR mediated by IFN, induce cell cycle arrest and apoptosis, further restricting the propagation of the virus. Furthermore, the innate immune response accelerates the clearance of OV via the activation of NK cells and the production of IFN-γ (186, 187). Simultaneously, DCs present viral antigens to CD4^+^ T cells, thereby initiating an antiviral immune response, while neutralizing antibodies produced by B cells further augment antiviral immunity (188). Although this antiviral response is effective in regulating viral dissemination and mitigating toxicity, it concurrently reduces the efficacy of OV-mediated tumor destruction. Contemporary approaches aimed at diminishing the clearance of viruses are diverse, including polymer encapsulation, the replacement of protein coronas, the utilization of nanovesicle shielding to circumvent neutralizing antibodies, serotype switching, and the implementation of cell-based delivery systems that extend viral activity for the advancement of antitumor immunity (85, 189, 190).

An alternative perspective posits that the antiviral response may yield beneficial effects. Gujar et al. illustrated that antiviral CD4^+^ T cells enhance the responses of tumor-specific CD8^+^ T cells (188). These CD4^+^ T cells facilitate the maturation of DCs through interactions involving CD40-CD40L and MHC II/epitope-TCR, which allows for the cross-presentation of tumor antigens to CD8^+^ T cells. Consequently, these CD8^+^ T cells are able to target and eliminate OV-infected tumor cells (191, 192). Furthermore, Zamarin et al. demonstrated that pre-existing immunity to NDV enhance its therapeutic efficacy by augmenting systemic antitumor immunity (193).

The dual characteristics of antiviral immunity, which both inhibit OV replication and enhance antitumor immunity, present a therapeutic paradox. Addressing this issue necessitates a comprehensive understanding of the interactions among viruses, tumors, and the immune system in order to optimize therapeutic opportunities.

### 7.3 Potential biomarkers

A significant obstacle in the translation of OV therapy from laboratory settings to clinical application is the absence of predictive biomarkers that are grounded in the viral mechanisms of action. In contrast to well-established biomarkers, such as PD-L1 expression and elevated tumor mutational burden (TMB), which are utilized in ICI therapy, research on OV biomarkers remains in its nascent phase. There exists an urgent requirement for systematic biomarkers capable of elucidating the intricate interactions among viruses, tumors, and the immune system.

In preclinical studies, a recombinant HSV-1 vector that expresses hPD-1scFv has been shown to upregulate CTLA-4 and TIM-3 on exhausted CD8^+^ T cells (194). An immunohistochemical analysis conducted on 19 biopsy samples indicated that TIM-3 expression was significantly elevated in patients with a poor prognosis (P = 0.006) (195). Conversely, a clinical investigation involving 15 patients with various cancer types revealed that 60% of these patients exhibited downregulation of TIM-3 expression, which was associated with markedly improved clinical outcomes. Mechanistically, the downregulation of TIM-3 facilitated the redistribution and infiltration of CD8^+^ T cells into the tumor core, thereby increasing TILs (196). These findings suggest that TIM-3 may represent a potential biomarker for OV therapy; however, further validation through additional clinical trials is warranted. Notably, investigations into YST-OVH have revealed that tumors exhibiting elevated immune activation at baseline are more prone to demonstrate immune suppression. Initial studies have identified several factors, including B cell activation, complement activity, tumor-associated macrophages (TAM), and IFN signaling pathways, as potential prognostic and predictive biomarkers for OV therapy (194).

Deficiencies in host antiviral mechanisms are increasingly recognized as potential predictive biomarkers for OV therapy. For instance, indicators associated with the IFN pathway, such as the characteristics of interferon-stimulated genes (ISGs) including MX1, EPSTI1, XAF1, and GBP1, have been correlated with tumor sensitivity to VSV (197). In a departure from conventional paradigms, Ishino et al. demonstrated that oncolytic HSV-1 represents a promising therapeutic approach for hematological malignancies. The expression of nectin-1, rather than deficiencies in the cellular antiviral mechanisms, is a critical determinant of tumor cell susceptibility to HSV-1 and may serve as a predictor of therapeutic efficacy (198). Additionally, another investigation revealed that D2HG, a metabolite produced as a result of IDH1 mutations, impedes the IFN antiviral response in glioma cells, thereby increasing their sensitivity to OV therapy (199). Moreover, immunoglobulin-like transcript 2 (ILT2), a significant inhibitor of T cell responses, may function as a potential biomarker for assessing clinical responses in melanoma patients undergoing treatment with VV (200). Moreover, fluctuations in the expression levels of viral receptors, processing enzymes, and genes critical for viral infection may lead to varying degrees of susceptibility among cancer cells to particular viral agents.

Alterations in peripheral blood counts present a more straightforward and expedited predictive approach. For example, a Phase Ia/Ib clinical trial demonstrated that baseline neutrophil levels could serve as a predictor for the response to OH2, an oncolytic virus derived from HSV-2, in patients with advanced melanoma (201). Furthermore, the TUNIMO Phase I trial revealed that a decrease in acute lymphocyte levels following TILT-123 therapy is associated with therapeutic efficacy in a cohort of 20 patients with advanced solid tumors (202). These findings suggest a practical and cost-effective method for monitoring the efficacy of OV. Future investigations should aim to further validate the significance of these biomarkers in relation to other OVs to facilitate broader applicability. Additionally, a clinical study involving 202 cancer patients treated with an OAd identified low baseline serum levels of high mobility group box 1 protein (HMGB1) as an independent positive prognostic and predictive factor for oncolytic immunotherapy in individuals with advanced cancer (203).

The advancement of biomarkers for OV therapy encounters distinct challenges, particularly due to the significant tumor heterogeneity and the variability in immune pathway activation induced by different viral strains. Existing research has predominantly involved diverse cancer patient cohorts, and predictive models tailored to specific tumor types are still rare. To enhance the precision and clinical efficacy of OV therapy, it is imperative that future investigations prioritize the design of clinical trials within more homogeneous patient populations.

### 7.4 The Interaction between OVs and microbiome

The interplay between OVs and microbiome represents a burgeoning area of investigation within the realm of cancer immunotherapy. This interdisciplinary domain integrates aspects of virology, microbiome research, and tumor immunology, thereby providing novel insights for the treatment of cancer.

The gut microbiome, recognized as one of the most intricate microbial communities within the human body, is instrumental in modulating antitumor immune responses. Relevant research indicates that specific compositions of the gut microbiota are significantly associated with the effectiveness of OV therapy. A healthy gut microbiome facilitates T cell recognition of tumor antigens, promoting the activation of cytotoxic CD8^+^ T cells. Probiotic bacteria including Bacteroides fragilis, Akkermansia muciniphila, and Bifidobacterium have been linked to enhanced responses to immunotherapy (204–206). In the Delta-24-RGDOX model, a high prevalence of Bifidobacterium was associated with improved survival outcomes, and the antitumor effects of Ad5D24-CpG were found to be partially reliant on the modulation of the gut microbiome (207). These observations imply that strategically altering the gut microbiome to favor a more “beneficial” bacterial composition may represent a novel therapeutic approach to augment and predict clinical outcomes for cancer patients receiving immunotherapy. Interventions such as dietary modifications, probiotic supplementation, or fecal microbiota transplantation enhance patient responses and increase therapeutic efficacy (208).

Recent investigations have elucidated a complex interplay among the gut microbiome, OVs, and the IFN system. IFN is the first line of defense against pathogens and functions as a potent immunostimulant. It possesses various roles, including antiviral activity, immune regulation, and antitumor effects. Research conducted by Yi et al. indicates that the IFN-I response, which is stimulated by the microbiota, can enhance antiviral immunity; however, excessive activation of this response may accelerate the clearance of OVs (209). Achieving a balance among these elements is essential for optimizing treatment strategies, particularly in the context of CRC. Unlike conventional viral delivery methods, the oral administration of RV not only engaged with the host immune system but also resulted in the secretion of IgA^+^ antibodies in the Peyer’s patch of the terminal ileum (210).

In contrast to the gut microbiome, the tumor microbiome (TM), encompassing the bacteria, fungi, and viruses found within tumor tissue, remains inadequately characterized. A recent review has outlined various strategies aimed at modulating the TM to improve cancer treatment outcomes (211). Wu and colleagues discovered that infection with Fusobacterium nucleatum in gastric cancer cells can attract tumor-associated neutrophils, which subsequently enhance the expression of PD-L1. This mechanism facilitates immune evasion and sensitizes the tumor to ICB, while potentially diminishing the immune-activating effects of OVs (212).

A growing body of research has indicated that the diversity and composition of the host gut microbiota are correlated with the effectiveness of immunotherapy and the occurrence of irAEs. This suggests the potential for utilizing microbiome as innovative biomarkers to predict patient responses to immunotherapy, as well as targeting microbiome as prospective anticancer agents, either independently or as adjuncts. An in-depth investigation into the mechanisms by which microbiome function will not only deepen our comprehension of tumorigenesis and its progression but will also elucidate the interactions between OVs and the microbiome. This enhanced understanding will serve as a basis for the formulation of more targeted and effective therapeutic approaches.

## 8 Discussion

OVs have emerged as a promising category of immunotherapeutic agents, exhibiting notable safety profiles and the capacity to ameliorate the immunosuppressive characteristics of the TME. OAds are particularly distinguished by their unique benefits, with over fifty clinical trials currently registered on ClinicalTrials.gov (refer to **Table 5**). A significant advantage of OVs, in contrast to conventional therapies, lies in their reduced systemic toxicity and enhanced tumor selectivity. Engineered OVs induce direct cytotoxic effects on neoplastic cells through targeted oncolysis, while simultaneously augmenting systemic antitumor immune responses. This process involves the activation of DCs and the proliferation of antigen-specific T cells, which contribute to the establishment of long-term immune memory. While the clinical effectiveness of OV monotherapy may be limited, the combination of OVs with ICIs has the potential to address both primary and acquired resistance, thereby improving therapeutic outcomes. Moreover, the exclusive use of ICIs may result in significant off-target organ damage and irAEs, including immune-mediated pneumonia, myocarditis, and thyroid dysfunction, which can lead to treatment cessation or even mortality. The localized expression of checkpoint modulators via OVs may mitigate systemic toxicity while preserving antitumor efficacy.

**Table 5:** Summary of the OAds has been completed or recruiting in ClinicalTrials.gov (excluded suspend, withdraw, terminated study status).

OAds	Combination	Tumor type	Status	Delivery	Phase	Identity
Ad-TD-nsIL12	/	Primary pediatric DIPG	Recruiting	IT	I/II	NCT05717712
LOAd703	Atezolizumab	Melanoma	Completed	IT	I/II	NCT04123470
	Gemcitabine nab-paclitaxel atezolizumab	Pancreatic cancer	Recruiting	Percutaneous injection	I/IIa	NCT02705196
	/	Pancreatic cancer Biliary cancer OC CRC	Completed	IT	I/II	NCT03225989
TILT-123	/	Advanced solid tumors	Recruiting	IT	I	NCT04695327
	Pembrolizumab, pegylated liposomal doxorubicin	OC	Recruiting	IT/PI	I/Ib	NCT05271318
	Avelumab	SCCHN and melanoma	Recruiting	IT	I	NCT05222932
	Pembrolizumab	NSCLC	Recruiting	IV/IT	I	NCT06125197
	/	Melanoma	Active, not recruiting	IT	I	NCT04217473
DNX2401	Temozolomide	Recurrent GBM	Completed	IT	I	NCT01956734
MSC-DNX-2401	Conventional Surgery	High-grade glioma	Recruiting	IA	I	NCT03896568
CG0070	/	High-grade NMIBC	Completed	IVE	II	NCT02365818
H101	PD-1 Inhibitor	MPM	Recruiting	IT/intrapleural injection	I/II	NCT06031636
	Camrelizumab	NMIBC	Recruiting	IVE	II	NCT05564897
	Sorafenib	HCC	Unknown status	IT	I/II	NCT05113290
	HAIC of FOLFOX	ICC	Recruiting	IT	I/II	NCT05124002
ICOVIR-5	/	Advanced or metastatic melanoma	Completed	IV	I	NCT01864759
CGTG-102	low-dose oral cyclophosphamide	Advanced cancers	Completed	IT	I/II	NCT01598129
Oncolytic MG1-MAGEA3 With Ad-MAGEA3 Vaccine	Pembrolizumab	NSCLC	Completed	IM	I/II	NCT02879760
VCN-01	Gemcitabine, Abraxane	Pancreatic cancer	Completed	IT	I	NCT02045589
	Gemcitabine, Abraxane	Advanced solid tumors	Completed	IV	I	NCT02045602
Recombinant Human Adenovirus Type 5	HAIC of FOLFOX	ICC	Recruiting	IT	I/II	NCT05124002
NG-350A	Check point inhibitor	Advanced or metastatic epithelial tumors	Completed	IV	I	NCT03852511
CAdVEC	HER2 specific CAR T cells	HER2 positive solid tumors	Recruiting	IT	I	NCT03740256
KD01	/	Cervical malignancies	Recruiting	IT	I/II	NCT06552598
Ad5-yCD/mutTKSR39rep-hIL12	/	Prostate cancer	Completed	intraprostatic injection	I	NCT02555397
	5-fluorocytosine (5-FC)	Pancreatic cancer	Completed	IT	I	NCT03281382
SynOV1.1	/	AFP positive solid tumors	Recruiting	IT	I	NCT04612504
ColoAd1	/	Colon cancer NSCLC Bladder cancer Renal cell	Completed	IV/IT	I	NCT02053220
NSC-CRAd-S-p7	Concomitant RT at a dose of 60Gy, chemotherapy with TMZ	Malignant gliomas	Completed	IT	I	NCT03072134
TS-2021	/	Malignant glioma	Recruiting	IT	I/II	NCT06585527
Celyvir	/	Metastatic and refractory tumors	Completed	IV	I	NCT01844661
Enadenotucirev	Capecitabine, Radiotherapy	Rectal cancer	Completed	IV	I	NCT03916510
AdAPT-001	Checkpoint Inhibitor	Sarcoma and refractory solid tumors	Recruiting	IT/IA	II	NCT04673942
Immunostimulatory Oncolytic Adenovirus	/	Pancreatic cancer Biliary cancer OC CRC	Active, not recruiting	IT	I/II	NCT03225989
ORCA-010	/	Prostate Cancer	Active, not recruiting	IT	I/IIa	NCT04097002
YSCH-01	/	Relapsed/refractory solid tumors	Unknown status	IT	I/II	NCT05180851
ONCOS-102 (CGTG-102)	Cyclophosphamide	Refractory injectable solid tumors	Completed	IT	I/II	NCT01598129
	Pemetrexed/Cisplatin	MPM	Unknown status	IT	II	NCT02879669
	Pembrolizumab, cyclophosphamide	Advanced or unresectable melanoma	Completed	IT	I	NCT03003676
BioTTT001	/	Malignant solid tumors	Not yet recruiting	IT	I	NCT06215846
	SOX, Toripalimab	Peritoneal metastases from gastric cancer	Not yet recruiting	IP	I/II	NCT06283121
	Toripalimab, Regorafenib	CRC	Not yet recruiting	hepatic artery infusion	I//II	NCT06283134
	/	Recurrent/​progressive high-grade glioma	Enrolling by invitation	IT	Ib/II	NCT06763965
OBP-301	Pembrolizumab	Solid tumors	Completed	IT	I	NCT03172819
	Pembrolizumab	Advanced gastric gastroesophageal junction adenocarcinoma	Completed	IT	II	NCT03921021
	/	Metastatic melanoma	Unknown status	IT	IIa	NCT03190824
Recombinant Human Adenovirus Type 5	PD-1	Melanoma	Enrolling by invitation	IV/IT	I/II	NCT05928962
Ad MAGEA3	MG1-MAGEA3, pembrolizumab	Histological subtype of squamous and non-squamous NSCLC	Completed	IM, IV	I/II	NCT02879760

IT, intratumoral injection; IV, intravenous injection; IA, intra-arterial injection; IP, intraperitoneal perfusion; IM, intramuscular injection; SC, subcutaneous injection; IVE, intravesical injection; DIPG, diffuse intrinsic pontine gliomas; SCCHN, squamous cell carcinoma of the head and neck; NSCLC, non-small-cell lung cancer; HCC, hepatocellular carcinoma; RT, radiotherapy; TMZ, temozolomide; OC, ovarian cancer; CRC, colorectal cancer; GBM, glioblastoma; NMIBC, non-muscle invasive bladder cancer; MPM, malignant pleural mesothelioma; ICC, intrahepatic cholangiocarcinoma.

Contemporary clinical research predominantly centers on assessing the therapeutic efficacy of OVs in individuals diagnosed with advanced or metastatic cancer. Subgroup analyses indicate that prior treatment history significantly influences prognosis; specifically, patients who have previously received sorafenib or surgical interventions tend to experience less favorable clinical outcomes, whereas those who have undergone ablation therapy may derive potential survival advantages (15). In light of these observations, the incorporation of OV combination therapy could be contemplated as a first-line treatment strategy for certain malignant tumor (213). Nonetheless, the precise efficacy and safety of this approach require validation through comprehensive clinical trials.

IT administration continues to be the primary approach for therapeutic administration; however, the difficulties associated with targeting diffuse metastases have prompted the exploration of alternative delivery methods. IV administration expands the potential applications of OV therapy, yet it faces challenges related to the presence of neutralizing antibodies. The previously mentioned oral formulation utilizing VSV has shown preclinical safety and efficacy in murine models of colon cancer and melanoma. These results have been linked to the modulation of the gut microbiome and the activation of T cells, mechanisms that do not directly oncolysis (210). This method of administration presents several advantages, including the simplification of procedural requirements, increased clinical feasibility, and improved patient recruitment and adherence to treatment protocols. Furthermore, Additionally, the combination of VSV with αPD-1 (L1) and/or αCTLA-4 antibodies shows potential for inducing durable protective immunity and enhancing treatment tolerance (210).

In the future, critical elements for the progression of OV therapy involve the implementation of multicenter clinical trials to substantiate its antitumor efficacy, the development of engineered viruses with multifaceted functionalities, and the investigation of enhanced combinatorial strategies with ICIs. These synergistic initiatives are essential for repositioning OV from a secondary option for late-stage patients to a primary modality in cancer treatment, thereby establishing it as a significant contributor to the field of tumor immunology.

## Glossary

OVs: Oncolytic viruses
OAds: Oncolytic adenoviruses
ICIs: Immune checkpoint inhibitors
ICD: Immunogenic cell death
TME: Tumor microenvironment
TAAs: Tumor-associated antigens
VV: Vaccinia virus
HSV: Herpes simplex virus
RV: Reovirus
MeV: Measles virus
NSCLC: Non-small cell lung cancer
PD-1/PD-L1: Programmed cell death receptor/ligand 1
CTLA-4: Cytotoxic T lymphocyte-associated protein 4
CAR: Coxsackievirus-adenovirus receptor
CR2: Conserved region 2
Rb: Retinoblastoma
RCD: Regulated cell death
DAMPs: Damage-associated molecular patterns
PAMPs: Pathogen-associated molecular patterns
TAAs: Tumor-associated antigens
CRT: Calreticulin
HSPs: Heat shock proteins
HMGB1: High-mobility group box 1
DC: Dendritic
NK: Natural killer
APC: Antigen-presenting cell
CTLs: Cytotoxic T lymphocytes
GM-CSF: Granulocyte-macrophage colony-stimulating factor
TNF-α: Tumor necrosis factor-alpha
IL-2: Interleukin-2
DIPG: Diffuse intrinsic pontine glioma
HCC: Hepatocellular carcinoma
mOS: Median overall survival
ARR: Ascites response rate
ACR: Ascites control rate
PSA: Prostate-specific antigen
IT: Intratumoral
IV: Intravenous
IP: Intraperitoneal
SC: Subcutaneous
IVE: Intravesical
IM: Intramuscular
NSCs: Neural stem cells
MSCs: Mesenchymal stem cells
RCC: Renal cell carcinoma
LAG-3: Lymphocyte activation gene-3
TIGIT: T cell immunoreceptor with immunoglobulin and ITIM domain
TIM-3: T cell immunoglobulin and mucin-domain containing-3
SIRPα: Signal regulatory protein alpha
ICOS: Inducible T cell costimulator
TLRs: Toll-like receptors
irAEs: immune-related adverse events
ICB: Immune checkpoint blockade
EMT: Epithelial-mesenchymal transition
TMB: High tumor mutational burden
PDAC: Pancreatic ductal adenocarcinoma
TNBC: Triple-negative breast cancer
MSS: Microsatellite stable
ORR: Objective response rate
SD: Stable disease
CR: Complete remission
NDV: Newcastle disease virus
T-VEC: Talimogene laherparepvec
PFS: Progression-free survival
VSV: Vesicular stomatitis virus
TILs: Tumor-infiltrating lymphocytes
CTLs: Cytotoxic T lymphocytes
PSA: Prostate-specific antigen
Tregs: Regulatory T cells
TCR: T cell receptor
IFN: Interferon
CRC: Colorectal cancer
ACT: Adoptive cell therapy
DDR: DNA damage response
TAM: Tumor-associated macrophages
ILT2: Immunoglobulin-like transcript 2
TM: Tumor microbiome
T-VEC: Talimogene laherparepvec
MDSCs: Myeloid-derived suppressor cells
IRES: Internal ribosome entry site
BCG: Bacillus Calmette-Guérin
shRNA: Short hairpin RNA
IL-6: Interleukin-6
IL-12: Interleukin-12
IL-15: Interleukin-15
scFv: Single-chain variable fragment
sdAb: Single-domain antibody
5-FU: 5-fluorouracil
ISGs: Interferon-stimulated genes
HMGB1: High mobility group box 1 protein.
